# Fúlvio Pileggi: Um Ícone da Cardiologia Brasileira

**DOI:** 10.36660/abc.20210461

**Published:** 2021-07-15

**Authors:** Protásio L. da Luz, Charles Mady, Roberto Kalil, Carlos Eduardo Rochitte

**Affiliations:** 1 Universidade de São PauloHospital das Clinicas Instituto do CoraçãoSão PauloSPBrasilUniversidade de São Paulo – Hospital das Clinicas Instituto do Coração, São Paulo, SP - Brasil; 2 Hospital do CoraçãoSão PauloSPBrasilHospital do Coração (HCor), São Paulo, SP - Brasil

**Keywords:** Cardiologia, História, Ensino, Pesquisa, Humanismo

**Carlos Eduardo Rochitte**

Neste editorial especial gostaríamos de prestar homenagens a um ícone da Cardiologia brasileira e responsável direto pelo desenvolvimento de uma das maiores instituições da Cardiologia mundial, o Instituto do Coração (InCor), do Hospital das Clínicas da Faculdade de Medicina da Universidade de São Paulo (USP). Nas palavras de 3 pessoas muito próximas a ele (Professores Kalil, Mady e Protásio da Luz), vamos relembrar sua trajetória e traços de personalidade tão característicos deste líder inconteste de uma geração de cardiologistas. Como residente do InCor, tive a honra de conhecer e interagir com Professor Fúlvio que teve papel fundamental na minha carreira acadêmica. Primeiro, uma aparente barreira para entrar na pós-graduação: “para se inscrever na pós tem que ter estágio fora do país”, associada ao fato de que quase todas as bolsas de estudo no exterior exigiam como pré-requisito estar inscrito em um programa de pós, pareceu colocar uma barreira intransponível. Mas na verdade só me estimulou ainda mais a procurar alternativas para transpô-la. E de fato, ele abriu todas as portas para que eu realizasse o estágio na Universidade de Johns Hopkins. Depois disso, minha jornada já era história. Uma dívida eterna e um aprendizado para vida.

Em nome do *Arquivos Brasileiros de Cardiologia* (*ABC Cardiol*) e da comunidade científica queremos agradecer a todas as contribuições que Professor Fúlvio, direta e indiretamente, fez para nossa ciência em Cardiologia. Apenas para breve contextualização, o Professor Fúlvio publicou no *ABC Cardiol*, como autor ou coautor, 341 artigos, sendo o InCor, hoje, a instituição que mais publica no periódico.

Mas vamos às palavras de quem realmente o conheceu nas suas mais diversas nuances.

**Protásio L. da Luz**

Nascido em São Carlos, Fúlvio José Carlos Pileggi,^1^ formou-se na Faculdade de Medicina da USP em 1947. Estagiou no Instituto de Cardiologia Ignacio Chávez, México, como pós-graduando em Cardiologia. Ao voltar ao Brasil fez toda carreira acadêmica na USP, sempre ao lado do Professor Luiz V. Décourt, a quem substituiu como Professor Titular de Cardiologia. Assumiu a Direção do InCor em 1982 permanecendo como seu Diretor até 1997. Várias facetas de seu caráter merecem destaque. Foi personalidade marcante, forte, crítico de ideias e com amplo interesse em esportes e música clássica; tornou-se grande apreciador de vinhos. Sempre se manteve fiel a seus inúmeros amigos com quem apreciava conversar e trocar ideias sobre os mais diversos assuntos. Suas amizades cobriam amplo leque: políticos, empresários, advogados, magistrados e companheiros da universidade, sem fazer qualquer distinção de ideologias, raça ou gênero. Era afável e respeitoso no trato pessoal.

Como médico, primou pelo conhecimento, bom senso e humanismo. De sólida formação clínica soube se cercar de especialistas nas novas áreas da Cardiologia, de modo que conseguiu aliar os aspectos humanos da profissão aos avanços das tecnologias de diagnóstico e tratamento. Íntegro, sempre colocou o interesse dos pacientes acima de tudo. Tratou igualmente personalidades importantes da vida nacional, incluindo presidentes da república, bem como homens simples do povo. Incansável estudioso mantinha-se constantemente atualizado. Graças a essas qualidades formou grande clínica particular, despertando irrestrita confiança em seus pacientes.

Como professor universitário dignificou como poucos a carreira acadêmica, priorizando competência, integridade e dedicação. Foi um dos que mais combateram a politização nas universidades, defendendo que o preenchimento de cargos acadêmicos, reitores por exemplo, fosse realizado entre acadêmicos e não por pessoas alheias aos princípios fundamentais da universidade. Buscou excelência profissional em todos os ramos da medicina. Formou inúmeros discípulos a quem estimulou constantemente mesmo depois de deixarem a USP. Singularmente, ensinou mais pelo exemplo do que pela retórica.

Como administrador fez do InCor-USP seu objetivo de vida. Ao criar a carreira de pesquisador, em tempo integral e com remuneração adequada, trouxe cientistas para a instituição tanto em áreas básicas como fisiologia, genética e biologia molecular, imunologia e biologia vascular, mas também para pesquisa clínica; isto mudou o patamar do InCor que passou de um hospital primariamente assistencial a um instituto de pesquisa e geração de conhecimentos. Era exigente como administrador, mas compreensivo, informal, íntegro e justo. Tinha clara visão do papel formador, desbravador, de uma instituição acadêmica. Assim, compreendeu e implementou o conceito sobre o qual o InCor se baseia em três pilares: ensino, assistência e pesquisa. No ensino, o InCor há muitos anos prepara especialistas do mais alto nível em cardiologia, pneumologia e cirurgia cardíaca e torácica, com seus cursos de mestrado, doutorado e especialização. De alto nível é seu curso de graduação, que dá a base que todo médico deve ter em cardiologia e pneumologia. Na assistência sempre procurou assegurar que o InCor prestasse assistência diagnóstica, terapêutica, psicológica, social, nutricional e outras, da melhor qualidade a todos os pacientes do SUS ou conveniados, independente de raça, gênero, ideologia ou religião. Através da Fundação Zerbini implementou vários programas de aperfeiçoamento institucional em áreas técnicas e humanas. Conseguiu a colaboração de inúmeros líderes da sociedade civil para a construção do InCor como entidade de excelência. O modelo de uma instituição público/privada serviu de inspiração para muitas outras instituições no país. O InCor pôde destinar, à época de sua chefia, 10% dos leitos para pacientes de convênios privados ou clientes particulares, que pagavam pelos bons serviços prestados pelos cardiologistas da instituição. Os outros 90% eram dedicados a pacientes do sistema público de saúde, que usufruíam da qualidade de ponta dos serviços do InCor, pela arrecadação obtida com convênios e particulares. Esse modelo gerencial flexível hoje se tornou uma referência para fundações e mantenedoras de hospitais pelo Brasil afora.

Recebeu inúmeros Prêmios e distinções: Membro Titular da Academia Nacional de Medicina, Prêmio Fundação Conrado Wessel além de várias distinções acadêmicas de outras universidades. O Centro de Pesquisas do InCor tem o seu nome.

Foi autor e co-autor de cerca de 480 artigos em revistas científicas nacionais e 233 em periódicos internacionais. Foi um visionário e conseguiu inspirar seus discípulos e colaboradores na missão de criar uma instituição – o InCor de alta qualidade e padrão internacional. Como ele mesmo declarou em seu último pronunciamento no InCor: “A chave da felicidade é sonhar. A chave do sucesso é tornar os sonhos uma realidade”.

A medicina brasileira se despede de um de seus maiores ícones. Fica o exemplo de uma vida plena de realizações para a humanidade. De Fúlvio Pileggi se pode dizer que lutou a vida inteira e, portanto, merece ser qualificado segundo Bertolt Brecht:

“Há homens que lutam um dia e são bons, há outros que lutam um ano e são melhores, há os que lutam muitos anos e são muito bons. Mas há os que lutam toda a vida e estes são imprescindíveis”.

**Charles Mady**

No InCor, na Faculdade de Medicina da USP, no Hospital das Clínicas e nas sociedades médicas e civis, perdemos Fúlvio José Carlos Pileggi, personagem histórico e eminente. Ele possuía uma personalidade única, transparente e sincera, gostassem ou não. É fácil profissionalmente descrever um acadêmico, com todas as suas publicações e sua experiência de ensino. É mais difícil comentar sobre o ser humano, suas visões e pensamentos, que certamente são seu maior legado e que o levaram a desenvolver nosso instituto após as administrações de Zerbini e Décourt. Não deve ter sido fácil suceder a tais gigantes, mas Pileggi conseguiu elevar ainda mais o InCor. Todos nós temos virtudes e defeitos, mas, nesta balança, as virtudes irrefutavelmente predominaram. Tive, juntamente com muitos outros, o privilégio de conviver com ele quase diariamente, acompanhando de perto sua atuação enquanto liderava o InCor e, antes disso, na Segunda Clínica Médica, como Professor Assistente do Professor Décourt.

Foi nesta ocasião, quando eu ainda era estudante do quarto ano, no ano 1968, que eu o conheci. Ele disse, brincando, que eu só andava com livro ou revista debaixo do braço. Passamos a discutir medicina com frequência e esse contato nos aproximou muito, mesmo durante o meu estágio, a residência e o ensino. Foi o período que mais aprendi e serviu de base para a minha formação como clínico geral e cardiologista. Os estudos de pós-graduação começavam no nível de mestrado e Pileggi me incentivou a realizar matrícula na primeira turma. Logo depois, ele me convidou para ser contratado como médico assistente e me colocou, junto com outros, no curso de doutorado; depois disso, nos acompanhou 24 horas por dia, querendo ser informado sobre o andamento de todas as atividades do instituto. Ele me instruiu a defender o ensino gratuito, sem que eu tivesse direto de argumentar com ele. Ele francamente me exigiu isso, e assim foi. Como não ser grato a essa pessoa que participou de forma tão ativa e construtiva na minha formação? Havia uma academia, uma equipe que, sob a sua orientação, levou o InCor ao auge, formando líderes interna e externamente, exigindo qualidade.

Ele tinha paixão também por outra academia, o Palmeiras.

Conhecia tudo e todos e era um grande líder. Ele exigiu resultados à sua maneira, amparado por uma equipe invejável, com Macruz, Tranchesi, Serro-Azul, Bellotti, Ebaid, Del Nero e muitos outros que estiveram ao seu lado. Sabia agregar quem produzia, conquistando o respeito de todos e valorizando o mérito. Ele era selvagem, como um bom descendente dos calabreses, gritando e esperneando, mas não era um homem de tristeza. Como um homem de sentimentos, superava qualquer agressividade. Naturalmente, nós tínhamos as nossas diferenças conceituais, mas foram logo resolvidas. A porta de seu escritório estava sempre aberta e recebia a todos mesmo sem hora marcada. Lagrimejavam-lhe os olhos quando ouvia seus tenores e barítonos italianos. Certa vez, em sua casa, ele chorou ao ouvir Giglio, após algumas taças de vinho, outra de suas paixões. Ele construíra uma adega invejável e era generoso em compartilhá-la. Em outra ocasião, durante um jantar em sua casa, na companhia de um pesquisador da Itália, ele nos ofereceu um Romanée-Conti e deu de presente uma garrafa de Château Lafite. Para os nossos almoços de quarta-feira, no restaurante Massimo, eu levava o melhor. Ele também era admirador da boa cozinha, e o anfitrião fazia questão de agradá-lo. Certa vez, relatou-nos emocionado que ao contar ao seu pai que havia passado no vestibular da USP em terceiro lugar, ele lhe perguntou, ‘Por que não entrou em primeiro?’ Exigências e exigente desde a infância.

As rédeas do InCor sempre estavam em suas mãos, mesmo nos finais de semana e feriados. Ele foi movido pela paixão. Pessoas influentes sempre foram incentivadas a fazer doações ao instituto. Certa vez, depois de um banqueiro ter sigo operado na sua sala, ele pediu uma determinada quantia para a compra de equipamento. O paciente hesitou e Pileggi disse-lhe que, como seu médico, exigia a quantia, porque o hospital salvara-lhe a vida. Naquele exato momento, ele ligou para o banco para autorizar a transferência.

Ele colocou o InCor acima de tudo, dizendo que ficava mais aqui do que em sua casa, porque também era sua casa. Este legado, este espírito acadêmico era, para mim, a coisa mais importante que ele adorava. Ele consistentemente usou sua considerável influência pelo bem desta casa. Ele raramente ficava desanimado em decorrência de algum acontecimento político e, quando isso acontecia, logo se levantava e voltava a produzir. Ele era avesso à mídia, nunca buscando sua atenção. Ao contrário, a mídia tentou alcançá-lo, sem sucesso.

No seu concurso para Professor Titular, o tema escolhido foi “Pericardites”, sobre o qual ele havia ministrado uma aula de mestrado. Ele se aposentou aos setenta anos, desgostoso com certos fatos. Com grande dificuldade, conseguimos trazê-lo de volta, reunindo velhos amigos e gerando alegria a todos.

Por tudo o que você conquistou, sempre terá um lugar de destaque na Medicina. Onde você estiver, saiba que o seu InCor guarda enorme respeito por você e você será sempre lembrado com saudade pelo inesquecível legado que deixou. Você fez a diferença, com coragem e determinação. Descanse em paz.

**Roberto Kalil Filho**

O Prof. Fúlvio Pileggi fez sua especialização em Cardiologia no México, voltou ao Brasil e tornou-se uma grande referência na Cardiologia do país durante décadas. Ele foi, em conjunto com o Prof. Zerbini e o com o Prof. Décourt, um dos idealizadores do InCor do Hospital das Clínicas da Faculdade de Medicina da USP. O Prof. Pileggi orientou e liderou o desenvolvimento do InCor, edificando a instituição que é parte da rede pública universitária voltada para o atendimento dos pacientes, ensino e pesquisa, embasados no humanismo. O InCor divulgou-se nas suas áreas de atuação e propagou ensinamentos e formação de competências para a sociedade. Tive o privilégio de testemunhar a contribuição do Prof. Pileggi, que aglutinava a ajuda de muitos dos seus pares professores e também da sociedade. Associou-se posteriormente o Prof. Jatene com a sua reconhecida contribuição.

A vida do Prof. Pileggi era este instituto.

Presença diária, constante, mesmo nos feriados e finais de semana, era atento a cada detalhe da instituição. Um dos cuidados do Prof. Pileggi era a incorporação tempestiva das tecnologias mais modernas e mais avançadas. Frequentemente, designava pessoas para conhecerem no exterior novos desenvolvimentos de tecnologia. Graças ao Prof. Fúlvio Pileggi, liderando colegas professores e outros profissionais possibilitou-se que o InCor alcançasse a excelência na assistência aos pacientes, ensino e pesquisa. Era um grande clínico, muito dedicado aos seus pacientes, atento na maior parte do tempo às suas necessidades. Eu fui assistente do Prof. Pileggi por longo tempo e pude acompanhar a preocupação em atender os pacientes continuamente. Ele estudava muito, diariamente até tarde da noite e acompanhava a literatura médica de modo muito assíduo. Foi uma liderança de alta estatura humana, médica e científica e continua vivo no legado que edificou e nos deixou.

Desse modo, o Prof. Pileggi permanece presente por meio de seus ensinamentos em todos nós. Nós o pressentimos em cada canto do hospital.

Permanece também em nós grande reverência e gratidão ao distinto ser humano e Professor de Medicina.

**Figura 1:**
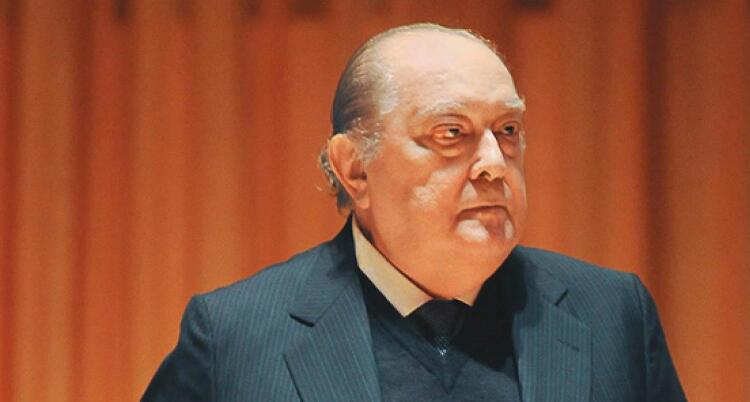
– Professor Fúlvio Pileggi fez do Incor - HC FMUSP seu objetivo de vida.
