# Supplementary material for: Fúlvio Pileggi: Um Ícone da Cardiologia Brasileira
Source: Arq Bras Cardiol. 2021 Jul 15;117(1):1–4. [Article in Portuguese] doi: 10.36660/abc.20210461 (PMC8294728; doi:10.36660/abc.20210461)
Supplement: Translation

# Translation: Fúlvio Pileggi: An Icon of Brazilian Cardiology

**Authors:** Protásio L. da Luz, Charles Mady, Roberto Kalil, Carlos Eduardo Rochitte

**Affiliations:** 1 BrasilUniversidade de São Paulo – Hospital das Clinicas Instituto do Coração, São Paulo, SP - Brasil; 2 BrasilHospital do Coração (HCor), São Paulo, SP - Brasil

**Keywords:** Cardiology, History, Teaching, Research, Humanism

**Carlos Eduardo Rochitte**

In this special editorial, we would like to pay tribute to an icon of Brazilian Cardiology who was directly responsible for developing one of the greatest cardiology institutions in the world, The Heart Institute (InCor), at the *Hospital das Clínicas* of the Faculty of Medicine of the University of São Paulo (USP). In the words of 3 people who were very close to him (Professors Kalil, Mady, and Protásio da Luz), we shall remember the career and the personality traits that characterized him as an undisputed leader for a generation of cardiologists. As a resident of InCor, I had the honor of meeting and interacting with Professor Pileggi, and he played a fundamental role in my academic career. First, there was apparently an obstacle to getting into a postgraduate program, “To enroll in a postgraduate program, you must have completed an internship abroad.” This, in association with the fact that nearly all scholarships to study abroad required, as a pre-requisite, that the applicant be enrolled in a postgraduate program, seemed to be an insurmountable barrier, which only served to stimulate me to look even harder for alternative ways to surmount it. And, in fact, he was the one who opened all the doors to make it possible for me to complete an internship at Johns Hopkins University. After that, the rest was history. I am eternally indebted to him for his teachings.

On behalf of the *Arquivos Brasileiros de Cardiologia* (*ABC Cardiol*) and the scientific community as a whole, we would like to thank Professor Pileggi for all the contributions that he made, directly and indirectly, to the science of Cardiology. To give a brief overview, Professor Pileggi published, as author or co-author, 341 articles in *ABC Cardiol*, and InCor is currently the institution responsible for the highest number of publications in this journal.

But let us hear from those who knew him best, in all of his diverse nuances.

**Protásio L. da Luz**

Born in the city of São Carlos, São Paulo, Fúlvio José Carlos Pileggi^1^ graduated from the USP Faculty of Medicine in 1947. He completed an internship at the Ignacio Chávez Institute of Cardiology in Mexico, as a postgraduate student of Cardiology. Upon his return to Brazil, he dedicated his entire academic career to the USP, constantly alongside Professor Luiz V. Décourt, whom he would eventually replace as Full Professor of Cardiology. He became the Director of InCor in 1982, a position which he would hold until 1997. Diverse facets of his character are worthy of mention. He had a strong, memorable personality; he was a critical thinker, with broad interests including sports and classical music; he became a notable wine connoisseur. He was constantly faithful to his countless friends, and he enjoyed exchanging points of view on any subject imaginable. His friendships included an array of politicians, businesspeople, lawyers, magistrates, and university colleagues, and he made no distinctions of race or gender. In all personal dealings, he was affable and respectful.

As a doctor, he excelled in knowledge, good sense, and humanism. With his solid clinical background, he knew how to surround himself with specialists from new areas of Cardiology, in a manner that successfully combined the human aspects of the profession with technological advances in diagnosis and treatment. Guided by integrity, he always placed his patients’ interests above everything else. He treated important Brazilian figures, including presidents, the same way he treated everyday citizens. As a tireless scholar, he was always up-to-date. Thanks to these qualities, he founded an important private clinic, gaining his patients’ unconditional trust.

As a university professor, he had an exceptionally distinguished academic career, giving absolute priority to competence, integrity, and dedication. He was one of the professors who fought hardest against the politicization of the universities, defending the idea that academic positions, for example, university deans, should be nominated by academic peers, rather than by individuals from the outside who did not participate in the university’s fundamental principles. He sought professional excellence in all branches of medicine, and he guided countless disciples whom he consistently stimulated, even after they graduated from USP. Singularly, he taught by example, more than by rhetoric.

As a manager, he made InCor and USP his life goal. By creating full-time, well-paid careers in research, he brought scientists to the institute, in both basic areas and in physiology, genetics, molecular biology, immunology, and vascular biology, in addition to clinical research. This took InCor to a new level. It went from a primary care hospital to a research institute that produced knowledge. He was a demanding administrator, but he was understanding, informal, upright, and fair. He had a clear vision of the academic institution’s formative, groundbreaking role. Thus, he understood and implemented the concept wherein InCor was based on three pillars: teaching, care, and research. With respect to teaching, InCor has, for many years, prepared specialists on the highest levels of Cardiology, Pneumology, and Cardiac and Thoracic Surgery, with its master’s, doctorate, and specialization courses. Its undergraduate course is high-ranking, and it provides the basis that every doctor should have in Cardiology and Pneumology. With respect to care, he always sought to guarantee that InCor would provide the highest quality of diagnostic, therapeutic, psychological, social, nutritional, and other forms of care to all patients, both those covered by Brazil’s public Unified Health System (SUS) and those with private healthcare plans, regardless of race, gender, ideology, or religion. Through the Zerbini Foundation, he implemented several programs in technical and human areas. He was able to guarantee support from diverse leaders of civil society in order to build InCor as an entity of excellence. The institute’s public/private model has been a source of inspiration to many other institutions throughout Brazil. At the time of his leadership, InCor was able to allocate 10% of beds to patients with private healthcare plans or private patients who paid for the excellent services provided by the institute’s cardiologists. The other 90% of beds were reserved for patients from the SUS, who received InCor’s state-of-the-art services, made possible by funds obtained from private healthcare plans and private individuals. This flexible management model has, today, become a reference for foundations and hospital managers all around Brazil.

He received countless awards and distinctions, including Full Member of the Brazilian National Academy of Medicine, the Conrado Wessel Foundation Award, and diverse academic distinctions from other universities. InCor’s Research Center was named after him.

He was author or co-author of approximately 480 articles in Brazilian scientific journals and 233 articles in international journals. He was a visionary who managed to inspire his disciples and collaborators in the mission of creating a high-quality, world-class institution: InCor. As he himself stated in his final speech at InCor, “The key to happiness is dreaming. The key to success is making dreams come true ”

While Brazilian medicine says goodbye to one of its greatest icons, his example of a life full of achievements on behalf of humanity remains. We can say of Fúlvio Pileggi that he fought throughout his entire life, and he, therefore, deserves to be described in the words of Bertolt Brecht:

“There are men who fight for a day, and they are good. There are others who fight for a year, and they are better. There are those who fight many years, and they are very good, but there are those who fight their whole lives, and they are indispensable.”

**Charles Mady**

At InCor, the Faculty of Medicine of USP, the *Hospital das Clínicas*, and medical and civil societies, we have lost Fúlvio José Carlos Pileggi, a remarkable, historical figure. He had a unique, transparent and sincere personality, whether other people liked it or not. It is easy to professionally describe an academic, with his publications and teaching experience. It is more difficult to comment on the human being, his visions and thoughts, which are certainly his greatest legacy and which led him to develop our institution after the Zerbini and Décourt administrations. It cannot have been easy to succeed two giants, but Pileggi managed to elevate InCor even higher. We all have virtues and defects, but, on this scale, virtues predominated overwhelmingly. I was, together with many, privileged to live with him on an almost daily basis, closely watching his performance as he led InCor, and, before that, at the Second Medical Clinic, as Assistant Professor to Professor Décourt.

It was on that occasion, as a fourth year student, in 1968, that I met him and, he said, jokingly, that I went everywhere with a book or magazine under my arm. We started to discuss medicine frequently, and this contact brought us very close, even during my internship, residency, and teaching. It was the period that I learned the most, and it built the foundation of my training as a general practitioner and cardiologist. Postgraduate studies started at the master’s level, and Pileggi encouraged me to enroll in the first class. Soon after, he invited me to be hired as an assistant doctor, and placed me, along with others, in the doctorate course; after that, he followed us around the clock, wanting to be informed regarding the progress of all activities at the institution. He instructed me to defend free teaching, without waiting for an answer. He simply told me to do it, and so I did. How can I not be grateful to someone who participated actively, constructively, in my training? There was an academy, a team that, under his guidance, led InCor to its peak, forming leaders internally and externally, demanding quality.

He was also passionate about another team, the Brazilian football club Palmeiras.

He knew everyone and everything, and he was a great leader. He demanded results in his own way, aided by an enviable team, with Macruz, Tranchesi, Serro-Azul, Bellotti, Ebaid, Del Nero, and many others standing by his side. He aggregated those who produced, receiving the respect of all, valuing merit. He was wild, like a good descendant of Calabrians, kicking and screaming, but he was not a man of sorrows. As a heartfelt man, he overcame any aggressiveness. We naturally had our conceptual differences, but they were soon resolved. The door to his office was always open, and he welcomed everyone without an appointment. He came to tears, listening to his Italian tenors and baritones. Once, at home, he cried when he heard Giglio after a few glasses of wine, another of his passions. He built an enviable wine cellar, and he was generous in sharing it. Another time, at dinner at his home, in the company of an Italian researcher, he offered us a Romanée-Conti and gave him a bottle of Château Lafite as a present. For our Wednesday lunches, at the Massimo restaurant, I brought the best. He was also an admirer of fine cuisine, and the host made a point of pleasing him. Once, he told us with emotion, his told his father that he had placed third in the USP entrance exam, to which his father replied, “Why weren’t you first?” Demands and demanding since childhood.

InCor’s reins were always in his hands, even on weekends and holidays. He was driven by passion. Influential people were always urged to make donations to the institute. Once, after a banker had undergone surgery in his office, he requested a certain amount for the purchase of equipment. The patient hesitated, and Pileggi told him that, as his doctor, he demanded the sum, because the hospital had saved his life. That very moment, he called the bank to authorize the transfer.

He put InCor above all else, saying that he stayed here more than at his home, because it was also his home. This legacy, this academic spirit was, for me, the most important thing he worshipped. He consistently used his considerable influence for the sake of this house. He was rarely discouraged as a result of any political events, and, when he was, he would soon get up and start producing again. He was averse to the media, never seeking their attention. On the contrary, the media attempted to reach him, without success.

In his competitive exam for Full Professor, the chosen theme was “Pericardites”, on which he had taught a master’s class. He retired at the age of seventy, disgusted with certain facts. With great difficulty, we managed to bring him back, reuniting old friends and bringing joy to everyone.

For all that you have accomplished, you will always hold a prominent place in Medicine. Wherever you are, know that your InCor has enormous respect for you, and that you will always be lovingly remembered for the unforgettable legacy you have left. You made a difference, with courage and determination. Rest in peace.

**Roberto Kalil Filho**

Professor Fúlvio Pileggi completed his specialization in Cardiology in Mexico. When he returned to Brazil, he became a major reference in Cardiology for decades. Together with Professor Zerbini and Professor Décourt, he was one of the founders of InCor at the *Hospital das Clínicas* of the USP Faculty of Medicine. Professor Pileggi guided and led the development of InCor, building the institute that is part of the public network of university hospitals dedicated to patient care, teaching, and research, based on humanism. InCor branched out into the areas in which it now acts, and it disseminated teaching and skilled training throughout society. I had the privilege of witnessing Professor Pileggi’s contributions, as he united his peers, professors, and society as well. He later reached out to Professor Jatene, with his well-known contribution.

This institute was his life.

As a daily and constant presence, even on weekends and holidays, he was attentive to every detail of the institute. One of Professor Pileggi’s concerns was the timely implementation of the most modern and advanced pieces of technology. He would frequently appoint people to go abroad to discover new technological developments. Professor Fúlvio Pileggi’s leadership of colleagues, professors, and other professionals, made it possible for InCor to achieve excellence in patient care, teaching, and research. He was an excellent physician who was very dedicated to his patients, and he was always attentive to their needs. As Professor Pileggi’s assistant for a long time, I was able to follow his concerns in continually providing care for patients. He studied hard, on a daily basis, until late at night, assiduously staying up-to-date on medical literature. He was an outstanding human being, doctor, and scientist, and he is still alive in the legacy that he built and left us.

In this manner, Professor Pileggi is still with all of us in his teachings. We can feel his presence in every corner of the hospital.

Our great reverence and gratitude to the distinguished human being and Professor of Medicine likewise endure.

**Figure 1:** – Professor Fúlvio Pileggi made Incor - HC FMUSP his life’s objective.
